# Panniculitis Ossificans in Posterior Knee: An Unusual Presentation

**DOI:** 10.7759/cureus.19369

**Published:** 2021-11-08

**Authors:** Fahad M Alkhuzaei, Rani Alsairafi, Khalid M Alqurashi, Rana S AL-Zaidi

**Affiliations:** 1 General Surgery, King Faisal Hospital, Makkah, SAU; 2 General Surgery, Faculty of Medicine, Umm Al-Qura University, Makkah, SAU; 3 Laboratory and Blood Bank, King Faisal Hospital, Makkah, SAU

**Keywords:** general surgery, pathology, heterotopic ossification, case report, panniculitis ossificans

## Abstract

Panniculitis ossificans (PO) is a heterotopic ossification, benign recurring lesion, presenting in a variety of presentations anywhere in the body. The condition can sometimes be mistaken for a malignant bone tumor or long list of other differential diagnoses which have been mentioned in our report and that may lead to unnecessary wrong management. We report a case of a patient with subcutaneous ossification in the posterior aspect of the left knee. PO was confirmed histologically showing subcutaneous fat necrosis associated with osteoid material.

## Introduction

Heterotopic ossification (HO) is a broad term and involved in many pathological and histological findings. Based on etiology, it can be classified to primary or secondary (more common), in which heterotopic ossification can present as an acute painful lesion or can be asymptomatic. Panniculitis ossificans is a rare, self-limiting form of heterotopic ossification that might involve the subcutis as a reaction to trauma or with unknown etiology. However, HO is a general non-specific pathological finding, while panniculitis ossificans is a specific pathological diagnosis. The condition is benign but sometimes be mistaken as malignant bone tumor.

## Case presentation

A 46-year-old male presented to an emergency department complaining of non-progressing left thigh swelling for two years discovered suddenly. He has no associated pain, limitation of activities, no history of discharge, or skin changes. The patient is diabetic and was on metformin and gliclazide. He didn’t seek medical advice prior to his current presentation. There is no history of trauma. No family history of the same compliant or previous history of other lumps in other regions was noted.

There is a well-circumscribed hard lesion in the posterior aspect of the left thigh. The swelling is about 5x6 cm in size, hard, superficial not attached to underlying muscle, and with no skin changes. There was no deep or superficial inguinal lymphadenopathy. No other abnormalities were detected on examination.

Ultrasound of the left lower limb

A well-defined subcutaneous hard lesion measuring 40x35x12 mm was seen on the posterior aspect of the left knee. The lesion showed ill-defined margins with multiple flecks of calcifications. However, no cystic areas and no evidence of increased color flow were noticed inside the lesion. Differential diagnosis includes post-traumatic, calcified fat necrosis, and less likely vascular malformation. No abnormal collections were noticed.

**Figure 1:**
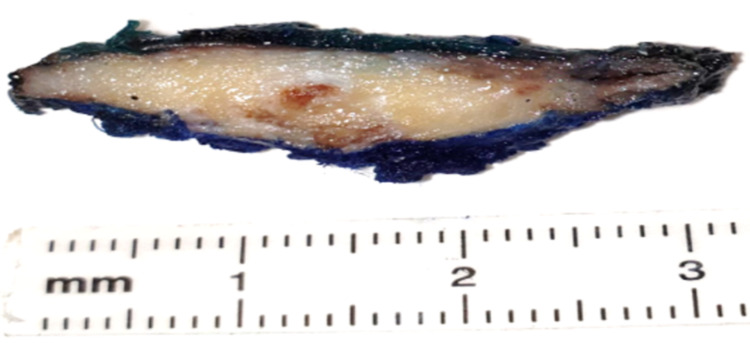
Gross image of panniculitis ossificans showing an ill-defined lesion at the center of the specimen comprised of a white glistening area at the top (represents the cartilaginous component) and a soft brown portion at the center (represents the fibro-vascular component).

Skin biopsy was performed and showed a small fragment of soft tissue consisting of interlacing bundles of spindle cells arranged in fascicles and admixed with fatty tissue exhibiting fat necrosis with fibrosis. Multiple fragments of ossification were noted. No abnormal mitoses were seen. The features were consistent with a solitary fibrous tumor. Pathological findings of the resected specimen fixed in 10% neutral buffered formalin revealed an irregular, tan-to-gray mass, and measuring 5x3x0.8 cm. Serial sections of the specimen revealed a heterogeneous tan/yellow-to-brown/white surface with a soft center and gritty sensation at the periphery (Figure 1).

**Figure 2:**
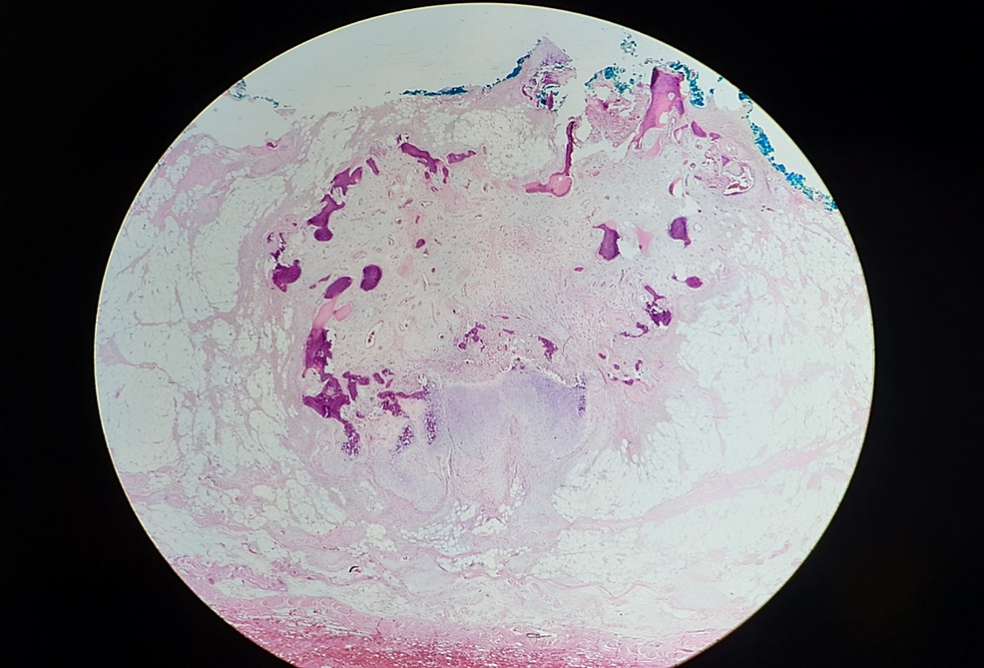
Panniculitis ossificans, a fairly circumscribed lesion in the subcutaneous tissue with distinct zonation. An inner zone of fibro-vascular tissue surrounded by an outer zone of metaplastic bone and cartilage is obvious here (hematoxylin-eosin, original magnification x20).

Microscopically, the lesion is comprised of an admixture of mature adipose tissue, bone, and granulation tissue-like fibroblastic proliferation with distinct zonation (Figure 2).

**Figure 3:**
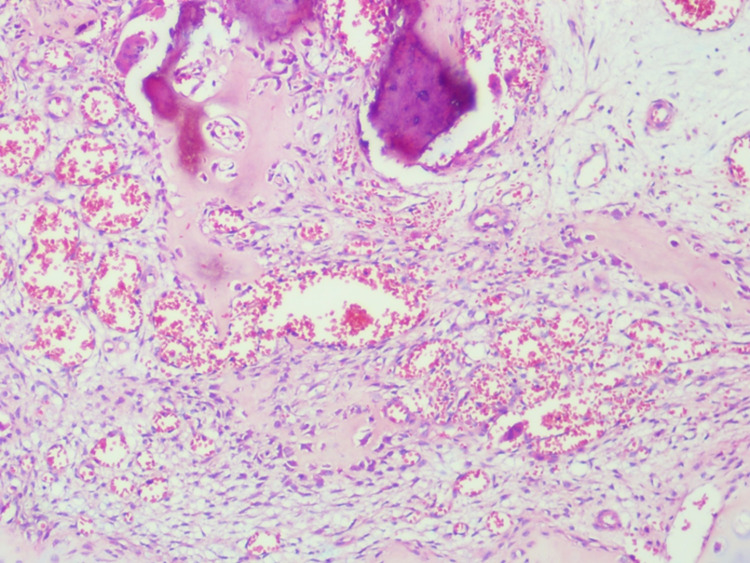
The inner zone of panniculitis ossificans showing loose myxoid stroma with prominent thin-walled ectatic blood vessels, hemorrhage, and reactive trabecular osteoid rimmed by plump osteoblasts (hematoxylin-eosin, original magnification x100).

The inner zone is comprised of fibroblastic/myofibroblastic proliferation of spindle to stellate cells with plump mildly pleomorphic nuclei exhibiting fine chromatin and prominent small nucleoli. The stroma was loose myxo-collagenous with prominent thin-walled small caliber to ectatic vessels, mild hemorrhage, scattered hemosiderin-laden macrophages, and scant inflammatory cells (Figure 3).

**Figure 4:**
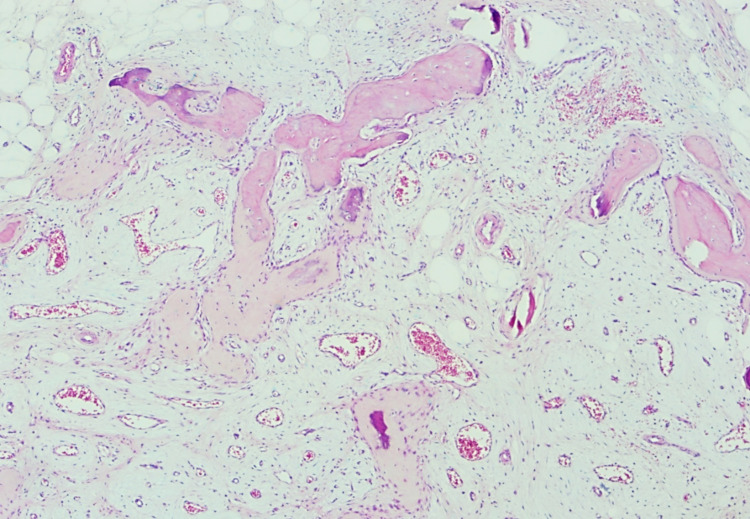
Progression of metaplastic bone formation from trabecular osteoid which is rimmed by plump osteoblasts into mature woven bone at the periphery of the lesion (hematoxylin-eosin, original magnification x40).

**Figure 5:**
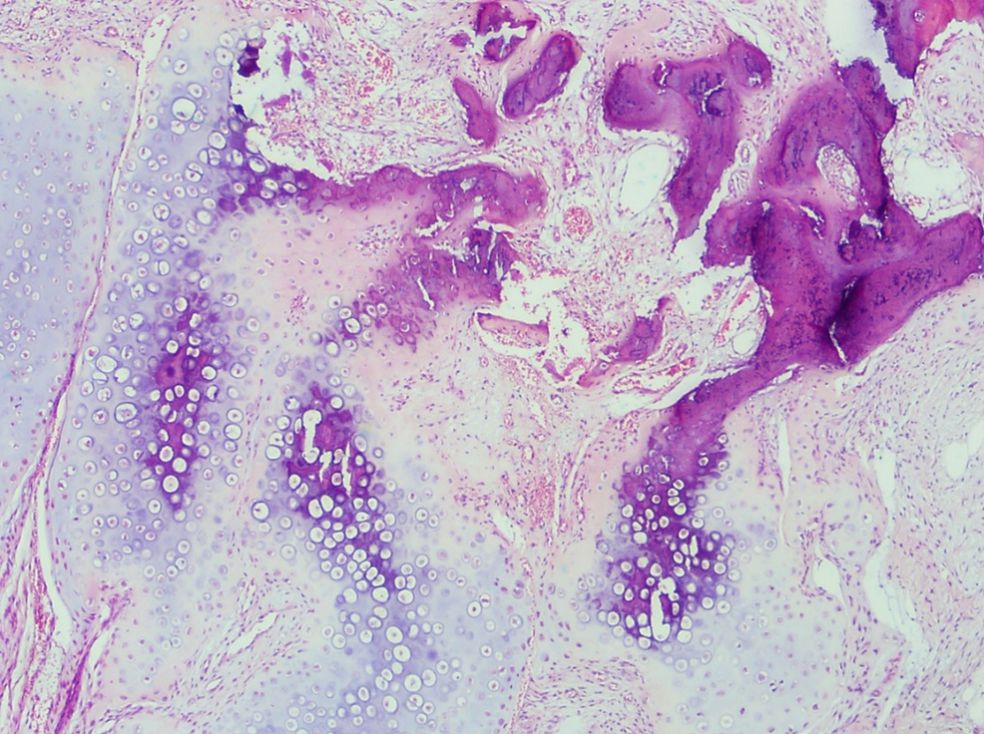
Panniculitis ossificans showing nodules of cellular hyaline cartilage undergoing endochondral ossification (hematoxylin-eosin, original magnification x40).

This is surrounded by a peripheral zone of trabecular osteoid, which is rimmed by plump osteoblasts and exhibits a progression into a variably mineralized trabecular woven bone than a well-formed mature lamellar bone (Figure 4). Nodules of variably cellular hyaline cartilage that undergoes endochondral ossification are noted as well (Figure 5). There was no marked nuclear atypia, atypical mitosis, or necrosis seen. Based on these histological findings, the final diagnosis is panniculitis ossificans.

## Discussion

Heterotopic ossiﬁcation (HO) near a joint was ﬁrst reported in the literature in 1978-1994. A total of four cases all at the distal phalanx were reported during this period. It is an uncommon disorder that consists of deposition of ectopic bone outside the extra-skeletal tissues [1]. The most common cause of HO, in general, is trauma, although, as in our case, atraumatic HO has also been reported. Trauma could be acquired as surgical, musculoskeletal, fight or warfare injuries. Other causes of HO are hereditary, burns, and neurogenic injury. Heterotopic ossification may occur in isolation or within dystrophic calcification and is distinguished by lamella architecture, osteocytes, and marrow formation (fatty and/or hematopoietic) [2].

Unlike calcification lesions, HO leads to a complete bone microenvironment within bone tissue cells, microcirculation, and neuroendocrine function. HO patients can experience pain and limited range of motion, which seriously impairs their activity of daily living [3]. Pathogenesis is not yet clear, although some studies found that it's multifactorial and the major factor identified is trauma.

The radiological findings of panniculitis ossificans are variable depending on the age and maturation of the lesion. In the early stages as in our case, plain radiography is negative due to lack of sufficient amount of calcification. However, ultrasound and magnetic resonance imaging with gadolinium contrast can diagnose and differentiate early cases of heterotopic ossification from sarcomas, as the pattern of enhancement is a rim in the periphery, while it is diffuse and homogenous in cases of malignancy [4].

Differential diagnosis depending on the clinical and radiological findings include nodular fasciitis, myositis ossificans progressive, myositis ossificans circumscripta, osteoma, chondrosarcoma, and osteosarcoma which should be excluded the most. Malignant fibrous histiocytoma, rhabdomyosarcoma, and slowly calcifying lesions like synovial sarcoma must be excluded as well.

As discussed earlier, patients may present with different complaints. Symptomatic treatment is recommended for HO in the formative stage that was revealed by bone scanning and radiographic examination while surgical excision can be done after complete maturation of bone. Stabilization and even regression have been reported before [5]. When HO is found in the skin it occurs in a variety of clinical settings and is called "osteoma cutis" [6].

HO is usually symptomatic, but it can be asymptomatic for years (as what happened with our patient). When symptomatic, it depends mainly on the size and anatomical site. Histologically, like in this case report, it's usually a lesion comprised of an admixture of mature adipose tissue, bone, and granulation tissue-like fibroblastic proliferation with distinct zonation. In some cases also defined as subcutaneous fat necrosis with a sequela of heterotopic bone formation. The condition is benign but can sometimes be mistaken for a malignant bone tumor [7].

## Conclusions

We conclude that panniculitis ossificans (heterotopic ossification in general) can occur in any age and with a wide variety of presentations. It can present anywhere as an asymptomatic silent lesion as in our case or acutely affecting activity of daily living. The crucial point in diagnosis is imaging and biopsy (after good history, examination, and exclusion of other differential diagnoses). The other anatomical presentations reported (e.g., mesenteric, neck region) have no alternative management rather than what already has been discussed in this report. Early diagnosis and management can prevent deformities and mobility restriction, which result in loss of function and esthetics.
